# Immunological age prediction in HIV-infected, ART-treated individuals

**DOI:** 10.18632/aging.203625

**Published:** 2021-10-11

**Authors:** Lesley R. de Armas, Suresh Pallikkuth, Li Pan, Stefano Rinaldi, Rajendra Pahwa, Savita Pahwa

**Affiliations:** 1 Department of Microbiology and Immunology, Miller School of Medicine, University of Miami, Miami, FL 33136, USA

**Keywords:** aging, inflammation, HIV, immune cells, biomarkers

## Abstract

Anti-retroviral therapy (ART) improves life expectancy in people living with HIV (PWH), but it remains unclear how chronic HIV infection affects normal aging of the immune system. Plasma cell-free protein expression and immune phenotypes were assessed in blood from ART treated PWH (19-77yrs, *n* = 106) and age-matched, HIV-negative controls (HC, *n* = 103). Using univariate spearman correlation, we identified 277 and 491 age-associated parameters out of a total 1,357 in HC and PWH, respectively. PWH exhibited shared and distinct age-associated immune profiles compared to HC highlighting the effect of HIV infection on immunological aging. Our analysis resulted in an 8-parameter, plasma-detectable inflammatory index that correlated with chronological age of all study participants but was higher overall in PWH. Additionally, predictive modeling for age in HC participants and age-associated parameters generated a 25-parameter signature, IMAP-25, with 70% and 53% accuracy in HC and PWH, respectively. Applying the IMAP-25 signature to immunological data from PWH revealed accelerated aging in PWH by 5.6 yrs. Overall, our results demonstrate that immune signatures, easily monitored in human blood samples, can be used as an indicator of one’s ‘immunological age’ during ART-treated HIV infection and can be applied to other disease states that affect the immune system.

## INTRODUCTION

The number of people in the US over 55 years of age living with HIV is on the rise, increasing from 27% in 2014 (of all diagnosed HIV infections in the US) to 35% in 2018 [1]. Anti-retroviral therapy (ART) has improved life expectancy for many people living with HIV (PWH), but this population exhibits a higher risk of age-associated comorbidities compared to young and middle-aged HIV negative populations [2]. Older PWH may be especially at risk since they are also less likely to have experienced the benefits of early initiation of ART (during acute infection) as guidelines have changed only within the last few years [3, 4]. Increased risk for co-morbidities is attributed to chronic activation of the immune system that persists despite viral suppression with ART [5–7]. On the other hand, higher levels of inflammation in advanced age can contribute to the pathogenesis of infectious disease, including HIV and tuberculosis, but also to cancer, diabetes, cardiac disease, and neurological complications [8, 9].

Age-induced decline in immune function is associated with mortality, however not all individuals age equally. Immune age is affected by one’s genetic background and environmental exposures throughout life [10, 11]. Studies performed in the general population (HIV uninfected) have identified signatures of aging from immunologic data including cellular phenotypes comprised of a loss of naïve T cells and CD38+ T cells with advanced age and an increase in CD57+ and effector memory T cells [12, 13]. Despite the natural variation in baseline immunological measurements that occurs on an intra- and inter-individual basis [14–16], the signatures were identified using cross-sectional analyses and confirmed in longitudinal analyses covering a nine-year window [13].

Based on epigenetic modeling using DNA methylation patterns in blood cells, PWH exhibit an increased aging rate compared to HIV negative individuals by an average of approximately 5 years with a 19% increased risk of mortality [17, 18], but how the epigenetic clock relates to immunological phenotypes and function is not known. Our previous studies have shown that age-correlated biomarkers in HIV-negative populations show weak associations with age in HIV positive groups [7, 19, 20]. Thus, additional models need to be generated to achieve a more comprehensive understanding of how the immune system reacts during advanced age in the context of HIV infection. Fortunately, many immune cells and immune signaling proteins are readily accessible from blood, thus providing an experimental framework for developing such models to compare immunological biomarkers during healthy and compromised states.

Here, we present a statistical strategy that generated immunological signatures from biomarkers easily monitored in human blood samples, that are predictive of biological age in adults. Using this strategy, we characterized features of the aging immune system that are altered during chronic HIV infection.

## RESULTS

### Inflammatory plasma protein profiles associate with age and HIV infection

**Figure 1:**
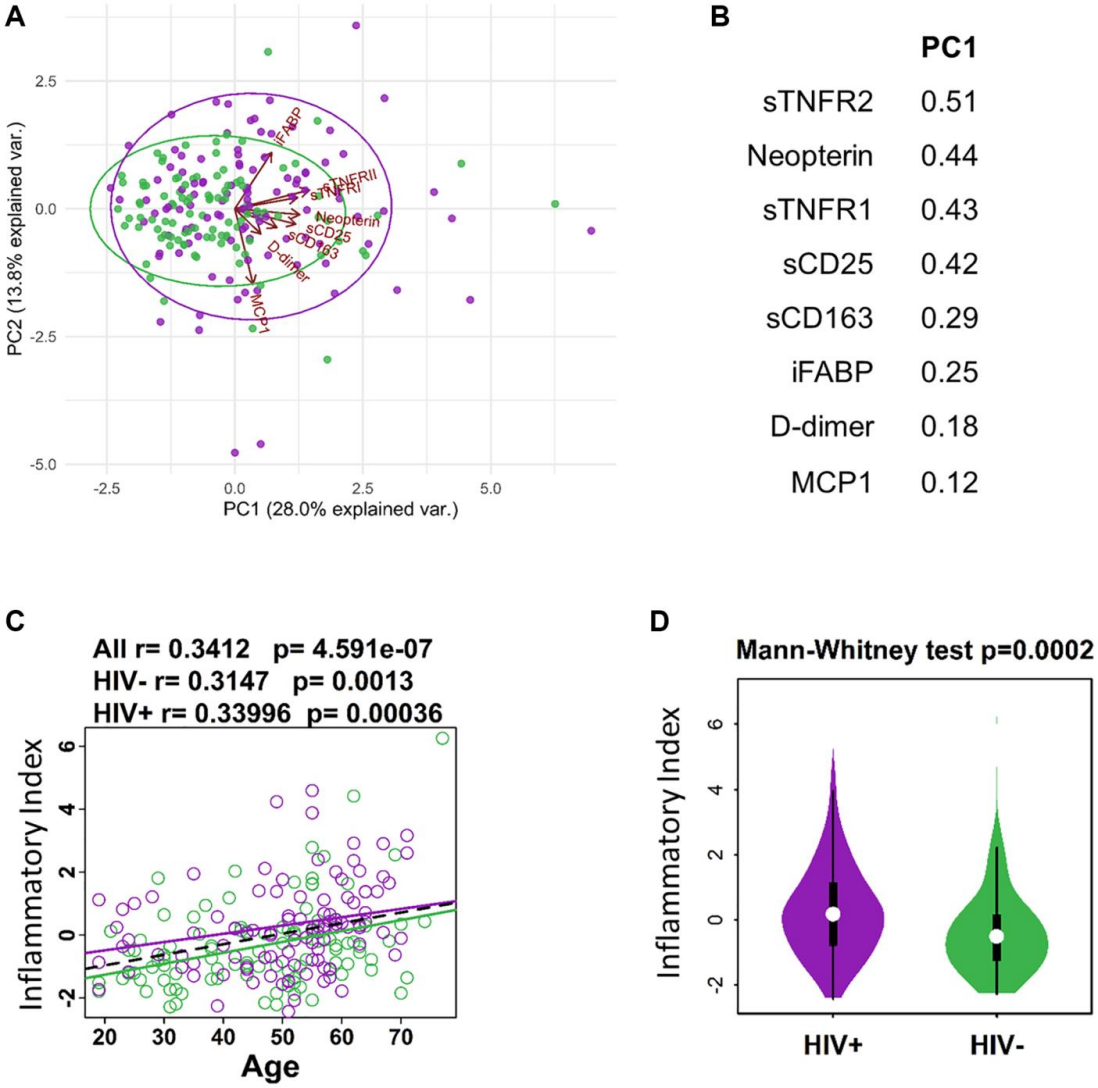
**Defining inflammatory index for aging and HIV infection.** (**A**) PCA plot showing distribution of HIV- (green) and HIV+ (purple) individuals in relation to protein expression from inflammatory index and Age in years. (**B**) List of proteins (standardized variables) in the inflammatory index and their weights extracted from PC1 in A. (**C**) Regression of Age with inflammatory index (sum of standardized variables from B multiplied by their weights extracted from PC1). (**D**) Violin plot showing the difference in inflammatory index between HIV- and HIV+ participants.

Inflammation and immune activation are independently associated with chronic HIV infection and an aging immune system. Thus, to study the effect of aging in the context of ART-treated HIV infection we performed the current study in participants from the Flu Responses of People in Relation to Age and HIV (FLORAH) study [19]. The age ranges of participating HIV-infected, ART-treated virally suppressed adults (*n* = 106) and HIV-negative, healthy controls (HC, *n* = 103) are shown in Table 1. Cell-free biomarkers associated with inflammation and immune activation (listed in Supplementary Table 1) were measured cross-sectionally from plasma collected at pre-vaccination in the related influenza vaccination study [7]. The panel of biomarkers selected for analysis were chosen to evaluate inflammatory cytokines and soluble receptors as well as markers of microbial translocation [21, 22]. Data reduction using Lasso was performed to generate an ‘Inflammatory Index’ containing only the biomarkers that most closely associated with age. This approach resulted in a group of 8 cell-free biomarkers with which the Inflammatory Index was calculated as the sum of the standardized variables of each biomarker multiplied with their weights extracted from principal component analysis variable PC1 (Figure 1A). Soluble TNF receptors 1 and 2 showed high contribution to the index, along with Neopterin, D-Dimer, soluble CD25 and CD163 receptors, MCP1, and iFABP (intestinal fatty acid binding protein) (Figure 1B). The index showed a significant but weak correlation (r value <0.4) with age in both HIV and HC (Figure 1C) and by comparing the groups regardless of age the index was higher in HIV compared to HC (Figure 1D). These results show that markers of inflammation that increase with age in the general population, are also higher in HIV-infected individuals supporting the hypothesis that HIV infection can enhance the process of inflammaging [23].

**Table 1:** FLORAH study participant demographics.

		**HIV Negative**			**HIV Positive**		
		**Young**	**Middle**	**Old**	**Young**	**Middle**	**Old**
*N*		35	49	18	20	61	25
Age Range (yrs)		19–39	41–59	60–77	19–39	40–59	60–71
Age Mean (yrs)		30.1	51.3	65.9	28.8	51.8	64.5
% Female		51%	47%	33%	50%	44%	28%
Duration of ART (years)	Mean + SD (*n*)	NA	NA	NA	5+/−2.8 (13)	10.8+/−5.7 (42)	12.9+/−7.6 (22)
Lymphocyte count	Cells/ul Mean (*n*)	2477 (33)	1904 (30)	1847 (17)	1754 (19)	1748 (37)	1692 (22)
CD4 count		987	999	871	784	727	596
CD8 count		395	325	432	458	366	527
**Race**	**Ethnicity**						
*White*	*Hispanic*	26%	31%	22%	25%	18%	24%
	*Non-Hispanic*	20%	14%	33%	0%	7%	8%
*Black*	*Hispanic*	6%	0%	11%	5%	5%	12%
	*Non-Hispanic*	29%	49%	17%	60%	69%	56%
*Asian*		11%	0%	17%	0%	0%	0%
*N.A.*		9%	6%	0%	10%	2%	0%
**Substance Use**	**Yes/No (%)**						
*Tobacco Use*		30/70	48/51	27/73	30/70	40/60	45/55
*Alcohol Use*		38/62	44/55	49/51	31/69	31/69	37/62

### Correlation of immunological parameters with chronological age

**Figure 2:**
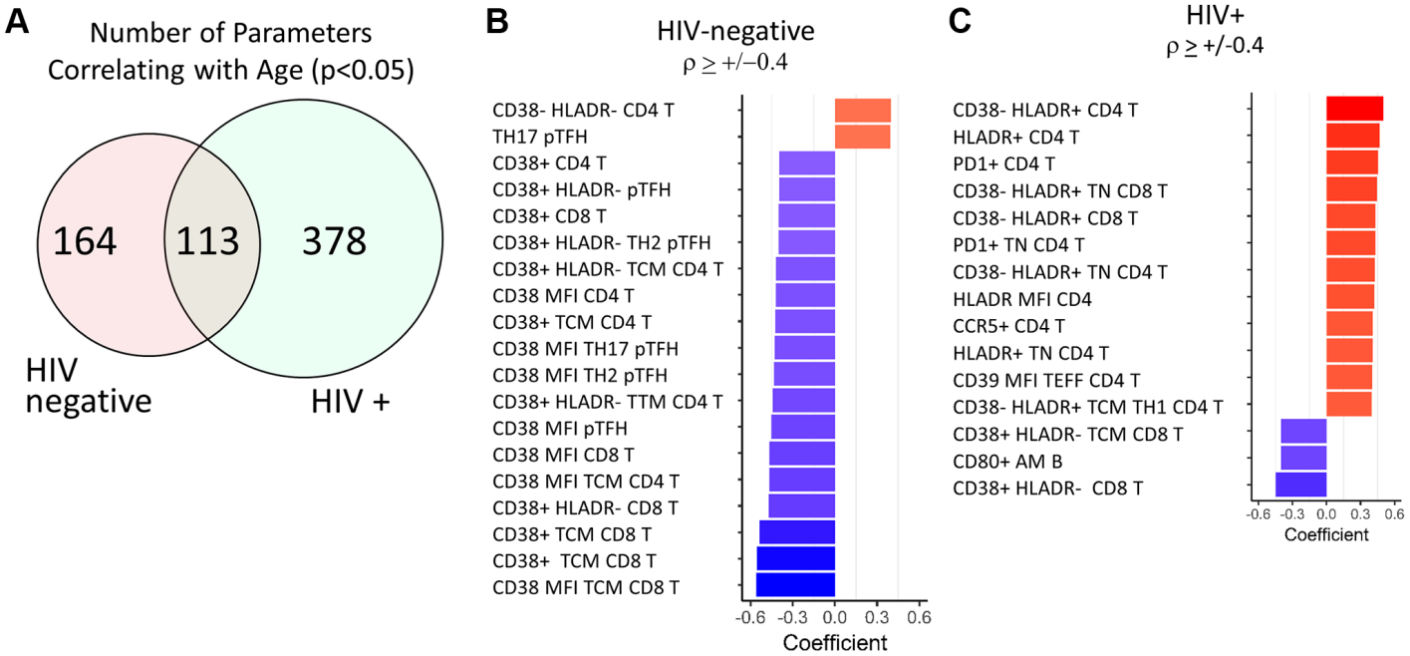
**Top age-associated immunological parameters in HIV-negative and HIV-positive study participants.** (**A**) Venn diagram showing the number of parameters with significant correlations with chronological age in HIV-negative (*n* = 103) and HIV-positive (*n* = 106) groups. Bar graph showing directionality of correlation coefficient for the top parameters with significant correlations with age in HIV-negative (**B**) and HIV-positive (**C**) participants. In red font are non-T cell parameters. Bold font indicates CD38 and HLADR containing parameters. Spearman test was performed for each parameter and chronological age, *p* < 0.05 was considered significant. In bar graphs, red denotes a positive correlation and blue denotes a negative correlation.

In pursuit of a predictive signature of age, we expanded the dataset to include flow cytometry-based phenotypic data acquired from the same individuals that were used to calculate the inflammatory index. PBMC samples collected prior to influenza vaccine administration were analyzed with 6 multiparameter, immunophenotyping panels that covered T cell, B cell, Monocyte, and NK cell frequencies and multiple phenotypic immune markers using 54 unique monoclonal antibodies (panels shown in Supplementary Table 2). This strategy generated a total of 1,357 parameters to interrogate expression patterns associated with age and HIV status. First, we wanted to establish an immune signature of aging in HIV-negative individuals as a reference population to test the hypothesis that HIV-infected individuals experience accelerated aging of the immune system. 277 parameters from the total 1,357 showed a significant correlation with age in HIV negative healthy controls (HC) when univariate spearman correlation analysis was performed (*p* < 0.05) (Figure 2A and Supplementary Table 3). Of these 277 parameters, 19 had correlation coefficients > +/− 0.4 and all 17 of the inversely correlated parameters were related to CD38 expression in T cells (Figure 2B). Both the frequency of total CD38 expression and mean fluorescence intensity (MFI) of CD38 was reduced with age in CD4 and CD8 T cells, including T Central Memory (TCM) subset and peripheral T follicular helper (pTfh) in CD4 T cells. The two parameters that demonstrated an increase with age were frequencies of pTfh TH17 cells and CD38-HLADR- CD4 T cells, the latter representing the opposite association compared to CD38+ T cells.

The same analysis was performed in the HIV-infected group of participants and 491/1,357 parameters showed a significant correlation with age (*p* < 0.05, Supplementary Table 4). Despite having ~2-fold higher number of correlating parameters compared to HC, only 15 parameters had correlation coefficients > +/−0.4 in the HIV group and most of these were T cell parameters (Figure 2C). However, the association patterns were strikingly different between the 2 groups. 8 out of 15 parameters included HLA-DR+ T cell frequencies or MFI expressed in Naïve and total CD4 and CD8 T cells and showed positive correlations with age. Expression levels of other known activation markers CCR5, PD1, and CD39 on CD4 T cells also increased with age in HIV. CD38 expression on CD8 T cells showed similar decreases with age as in HC. The only non-T cell parameter present in these results was the frequency of CD80+ activated memory (AM) B cells which also showed a negative association with age.

### Contrast of age-associated immunological parameters in PWH

**Figure 3:**
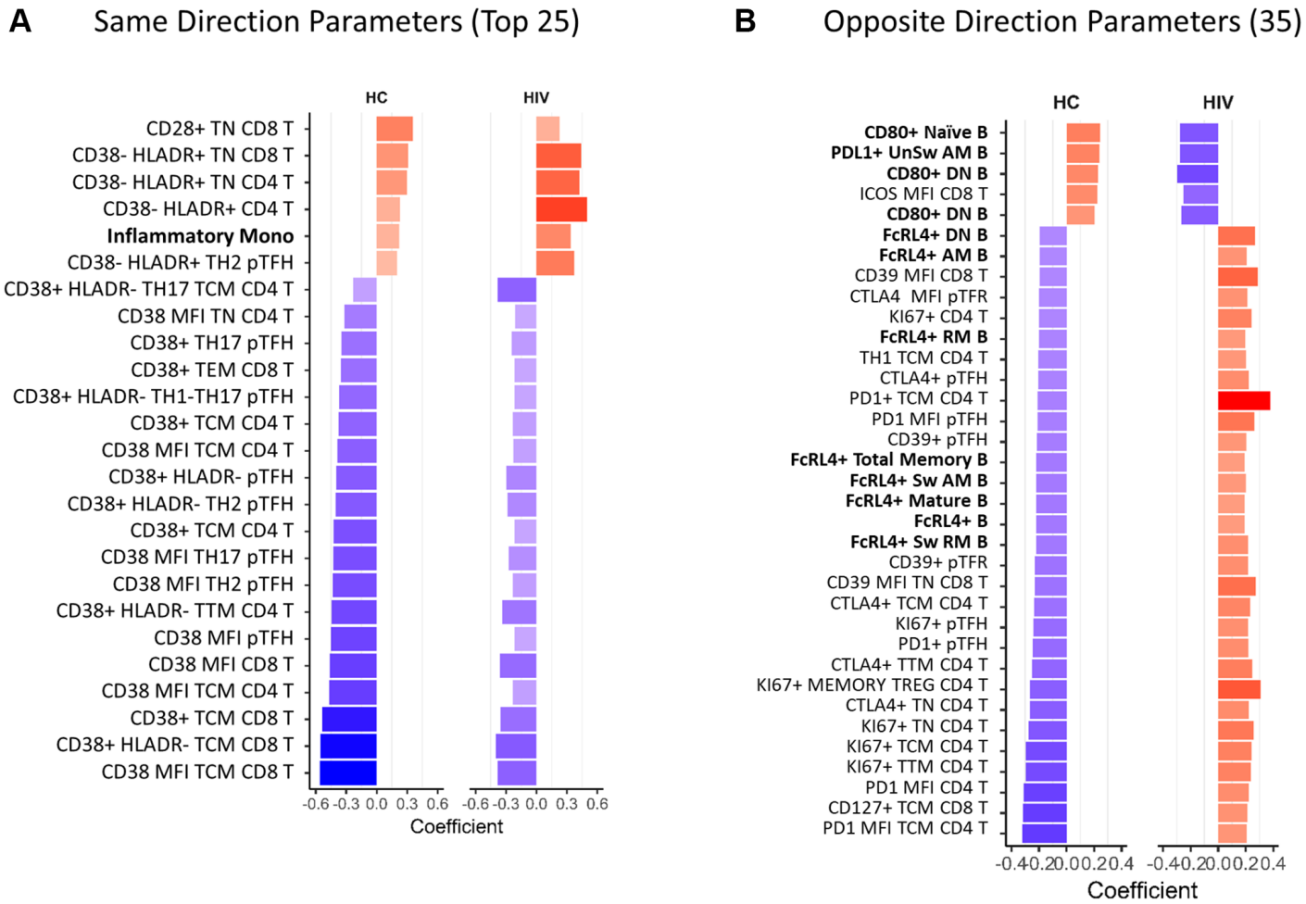
**Overlapping age-associated parameters in HIV-negative and HIV-positive groups.** Bar graphs showing correlation coefficients for the top parameters with same direction (**A**) and opposite direction (**B**) significant correlations with age in HIV-negative and HIV-positive participants. Only the top 25 out of 78 same direction parameters are displayed in (**A**), the rest are listed in Supplementary Table 3. All opposite direction parameters are shown in (**B**). In red font are non-T cell parameters. Bold font indicates CD38 and HLADR containing parameters. HC = HIV negative, healthy control. Spearman test was performed for each parameter and chronological age, *p* < 0.05 was considered significant. In bar graphs, red denotes a positive correlation and blue denotes a negative correlation.

To further understand the effect of HIV infection on aging of the immune system we compared all immune parameters that significantly correlated (*p* < 0.05) with age in both HC and HIV. 113 parameters overlapped in the two independent analyses (Figure 2A) and out of these, 78 showed the same direction of correlation in HC and HIV (Supplementary Table 5). Of the top 25 parameters with the strongest age association in both groups (Figure 3A), the majority of same-direction parameters included those with inverse correlations between CD38 expression on T cells and aging (48 out of 78 parameters). HLA-DR expression in certain T cell populations also showed increases with age in both groups. Additionally, frequencies of inflammatory monocytes showed a positive correlation with age in both groups.

The remaining 35 parameters had opposite directionality in association with age in HC and HIV (Figure 3B). In B cells, frequencies of CD80+ cells increased with age in HC and decreased in HIV, meanwhile FcRL4+ B cells decreased in HC and increased in HIV. In T cells, the markers that were differentially associated with age were CTLA4, PD1, and Ki67 expressed in CD4 T cells. These markers decreased with age in HC and increased in HIV. Overall, the univariate analysis demonstrated that T cell phenotypes, especially CD38 expression, that correlate with age in HIV-negative population are maintained in HIV+ individuals on ART though at a lower magnitude. While certain parameters with opposite associations with age in the HIV population reflect an altered aging pattern and may be important biologically.

### Prediction modeling for chronological age in HIV-negative population

**Figure 4:**
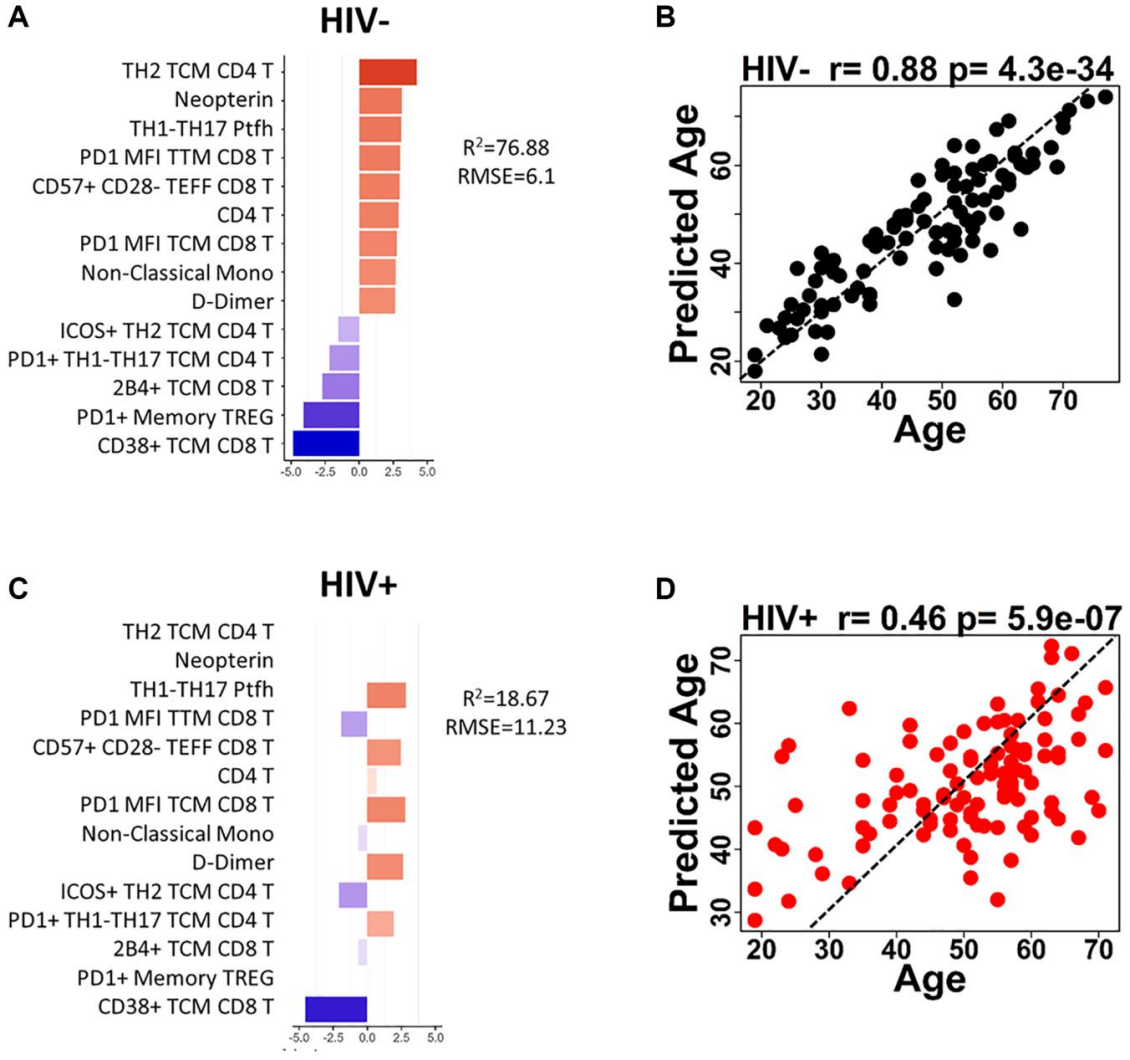
**Age prediction modeling using HIV-negative as reference population and age-associated parameters.** (**A**) Bar graph shows each of the 14 parameters included in the HIV-negative, trained model and indicates the coefficient for each parameter when applied to HIV negative participants. (**B**) The correlation between predicted age and actual age using the 14-parameter model is shown for HIV-negative participants. (**C**) Bar graph shows each of the 14 parameters included in the HIV-negative, trained model and indicates the coefficient for each parameter when applied to HIV positive participants. (**D**) The correlation between predicted age and actual age using the 14-parameter model is shown for HIV-positive participants. Correlations were determined using Spearman test. Red bars denote a positive association with predicted age and blue bars denote a negative association. Dotted lines in (**B** and **D**) show theoretical relationship for a perfect positive correlation for visualization purposes.

The univariate analysis for correlation between immunological parameters and age did not reveal a strong individual predictive biomarker for aging (the strongest correlation coefficient was −0.56 for CD38 MFI TCM CD8 T in HIV-negative). Thus, we combined all age-associated parameters to build prediction models for age in the participant groups with the goal of defining a small set of markers that could be used to predict the immunological age in PWH independent of chronological age. Significant age-associated markers (*p* < 0.05) were selected for the model using regression analyses Lasso or Elastic Net that perform regularization to reduce co-correlating parameters and overfitting of the model. The models were tuned to obtain the best prediction using 70% train and 30% test strategy first with HIV-negative participants. We found linear regression generated the best prediction among 4 machine learning algorithms (Linear regression, Neural network, Decision Forest regression, boosted decision tree). The modeling resulted in a 14-parameter model which had moderate predictive power with an R^2^ = 76.88% and root mean square error (RMSE) of 6.1 in HIV-negative (Figure 4A). The 14 parameters consisted of 9 parameters with a positive association with age and 5 with a negative association in HIV-negative participants. Cell-free markers D-Dimer and Neopterin were positively associated with age in the model. Total CD4 T cell frequencies and subsets of CD4 TCM Th2 and pTfh Th1/17 all showed positive associations with age. PD1 expression showed opposite effects on age depending on the type of T cell with expression on CD4 T cell subsets correlating positively with age in the model while PD1 on CD8 T cells decreased with age. CD38 expression in CD8 TCM had a negative association with age, as expected from the univariate analysis. Finally, frequencies of non-classical monocytes and immune senescent (CD57+CD28-) CD8 TEff cells were increased with increasing age. Predicted age and actual age showed strong correlation (r = 0.88, *p* = 4.3 × 10^−34^) as demonstrated by the good predictive accuracy in HIV negative participants (Figure 4B). When the HIV-negative (HC)-trained model was applied to the HIV group, it failed in predicting chronological age exhibiting extremely low predictive power at 18.67% (RMSE 11.23) and supporting the hypothesis that HIV infection alters the process of immunological aging (Figure 4C). Only 6 out of the 14 parameters maintained the same relationship with age in both HIV-negative and HIV-positive. Of note was the lack of association between frequencies of TCM/Th2 and Memory Treg/PD1 in CD4 T cells and Neopterin as a plasma marker. Although the 14-parameter model was not predictive in the HIV group the predicted age still showed a significant moderate correlation with actual age (r = 0.46, *p* = 5.9 × 10^-7^) (Figure 4D). Interestingly, this model revealed that many of the young HIV+ participants (<40 yrs) were being classified at higher ages, while older individuals (>50 yrs) were more likely to be accurately classified or even classified as younger than their actual age.

The same procedure was performed to determine whether a different set of immunological parameters could be used to predict age in the PWH group with greater accuracy. The modeling generated a 15-parameter model to predict aging in HIV, however it was weakly predictive with an r^2^ of 55.44% and RMSE 8.4 (Supplementary Figure 1).

### Age prediction modeling to determine aging rate in PWH

**Figure 5:**
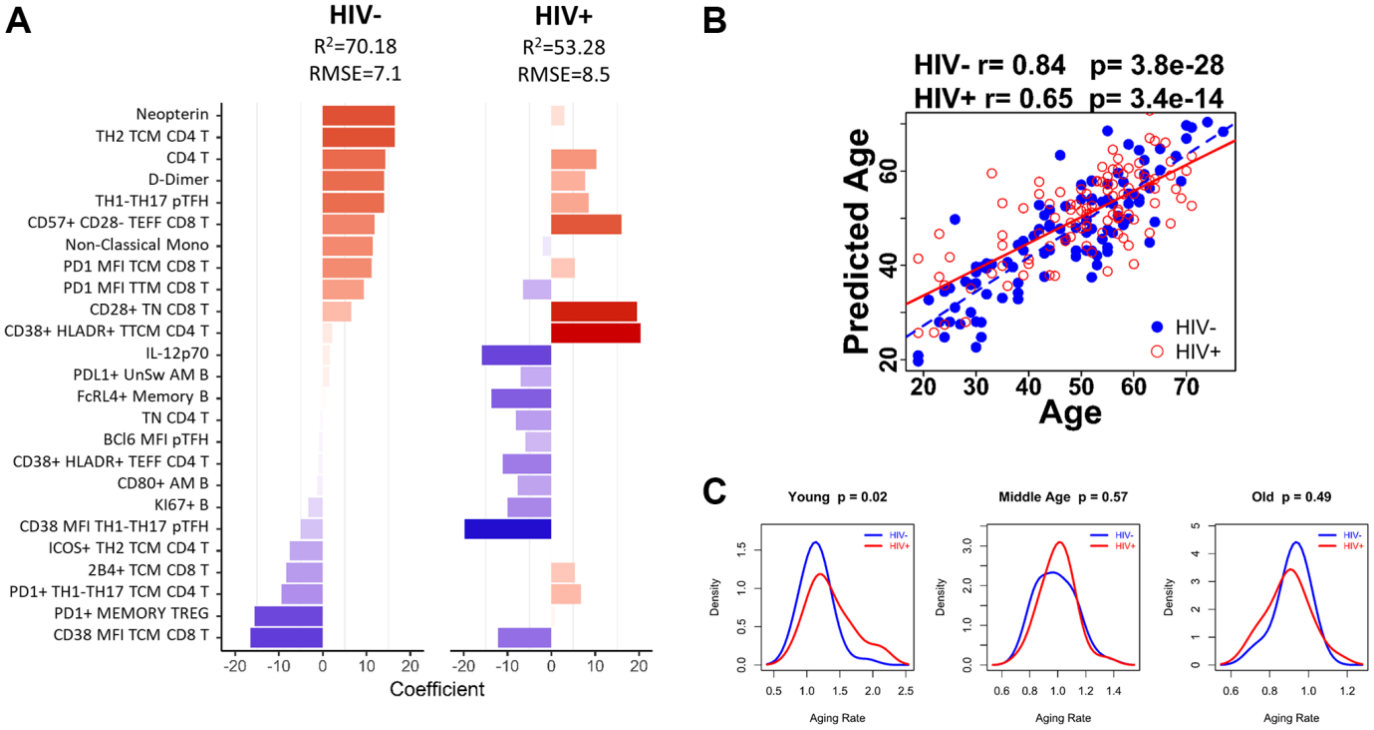
**Age-associated immune parameters reveal accelerated aging in young HIV-infected group.** (**A**) 25-parameter model to predict age in HC and HIV. Bar graph shows each of the parameters included in the model and indicates the coefficient for each parameter when applied to HIV negative (HC) participants and HIV-positive participants. Red bars denote a positive association with predicted age and blue bars denote a negative association. (**B**) The correlation between predicted age and actual age using the 25-parameter model is shown for HIV-negative (blue dots) and HIV-positive participants (red circles) using Spearman correlation test. (**C**) Density curves comparing the aging rate in HIV-negative (blue line) and HIV-positive (red line) participants in 3 age groups: Young (<40 yrs), Middle Age (41–59 yrs), and Old (>60 yrs). Student’s *t* test was performed to determine difference in aging rate, significance was considered when *p* < 0.05.

Combination of all parameters from the HC-trained model and HIV-trained model allowed us to generate an alternative model to predict age in both participant groups (Figure 5A). The results from the combined model highlight the difference in markers that associate with age in HIV. For example, CD38+ HLADR+ TTM CD4 T cell frequencies were positively associated with age in HIV but showed minimal association in HC. PD1 expression on T cell subsets showed stronger associations with age in the model for HC, but weakened or opposite associations in HIV.

The predicted age from the 25-parameter model called IMAP-25 (IMmunological Age Prediction) was strongly correlated with age in HC (Spearman r = 0.84, *p* = 3.8 × 10–28), and moderately correlated with age in HIV (Spearman r = 0.65, *p* = 3.4 × 10–14) (Figure 5B). However, the aging rate (calculated as the predicted age/actual age) was not significantly different in HC and HIV groups overall. Therefore, we divided the participants into age groups to perform additional analyses using the following criteria: <40 yrs (Young), 40–59 yrs (Middle), >60+ yrs (Old). We found that young HIV exhibited a faster aging rate than young HC by 18.47% (Welch Two Sample *t*-test *p* = 0.02), while Middle and Old aged participants showed similar aging rates in the two groups (Figure 5C). The increase in aging rate in young HIV corresponded to an age advancement of 5.62 years.

## DISCUSSION

In this study we applied a statistical analysis pipeline to evaluate a complex immunology dataset generated from human blood samples collected cross-sectionally to evaluate biomarkers of aging. Here, we used R software and established machine learning algorithms to compare and contrast patterns of expression of immunological biomarkers in Young and Old adults to determine a signature of aging and to allow us to answer the primary question of this research: How does chronic HIV infection affect normal aging of the immune system? The results show that HIV-infected, ART treated participants had many more differences than similarities in immune parameter expression patterns from early to elderly adulthood when compared to HIV-negative, healthy controls. This observation points to two possible explanations; first, that HIV infection changes immune parameter expression overall such that the patterns observed in aging are different, or second, that HIV infection influences immune parameter expression at the time of infection (usually in younger individuals) which in turn affects the overall pattern of parameter expression during aging. Predictive modeling using age-associated parameters and evaluation of aging rate in different age groups of HIV-infected and age-matched HC led us to conclude that the latter explanation is more accurate. The differences that we observed in age-associated parameters could be mostly attributed to differences observed between Young (<40yrs) participants with Young HIV exhibiting an accelerated rate of aging while Middle and Old participants had similar aging rates as HC groups.

The rate of age acceleration in the HIV-infected group in our study is strikingly similar to the age advancement rate determined in two independent studies that used DNA methylation to assess biological age (as opposed to the chronological age). Horvath, et al. developed an epigenetic clock using the extent of DNA methylation of a particular cell type or tissue to determine biological age and in multiple datasets using blood samples determined that HIV+ individuals showed a 5.2 yrs age advancement in biological age compared to their chronological age [17]. A separate study by Gross, et al. also using epigenetic models of biological age showed that age advancement in HIV-infected cohorts was an average of 4.9 yrs compared to HIV uninfected cohorts [18]. This study also suggested that HIV-infected people experience precocious aging as a consequence of acute infection rather than prolonged HIV infection since duration of infection had no impact on rate of age advancement. We did not have access to clinical data on duration of infection or CD4 nadir for the HIV-infected ART treated participants so we could not evaluate the contribution of these parameters to the immune signature that we defined. These parameters have been shown to contribute to higher levels of immune activation and inflammation during ART in some individuals [24, 25]. Overall, the concordance between our study and others in the literature is encouraging, however the relationship between DNA methylation and the immune parameters in the IMAP-25 signature are not understood. However, altered methylation patterns of the HLA locus has been documented in HIV [18, 26] which may explain the increased HLA-DR expression we observed with age in the HIV-infected participants only.

Further, precocious immune aging in ART-treated, HIV infection has biological relevance for immune function as was demonstrated in the influenza vaccine study using the FLORAH cohort [19]. Serum antibody titers against H1N1 and B strains that were included in the vaccine were much lower in Young (<40 yrs) HIV+ compared to Young HC post-vaccination. Old (60+ yrs) HC and Old HIV, however, generated similar levels of serum Ab titers to the vaccine though both groups had less than their Young counterparts.

Plasma biomarkers provide a snapshot of the level of systemic inflammation in the host and the literature has shown that levels of inflammation, as measured by IL-6, TNF, CRP, D-Dimer and others [27–30], are increased during HIV infection. Our analysis of only plasma biomarkers revealed a group of 8 cell-free proteins that formed an inflammatory index that increased with age in HIV and HC and was statistically higher in HIV compared to HC confirming the original hypothesis that HIV infection enhances the inflamm-aging phenotype. The markers in the index included soluble TNF receptors (sTNFR1 and sTNFR2) which result from shedding of the receptor from the membrane following TNF signaling and/or alternative splicing of the receptor [31] and have been associated with disease progression in HIV and aging previously [32–34]. MCP-1 is a biomarker related to neurocognitive dysfunction in people living with or without HIV [35] and also increases with age [7]. Additional markers in the index included soluble IL-2Rα (CD25), soluble CD163, neopterin, and D-dimer which have all been shown to be biomarkers for several inflammatory diseases indicating poor prognosis [36, 37]. The intestinal Fatty Acid binding protein (iFABP) is a marker of microbial translocation and has been shown to remain elevated in HIV-infected individuals with ART [38, 39]. A recent study has presented REG3α as a novel marker for gut damage in PWH that may be more informative than iFABP in assessing microbial translocation [40], however its relationship to Age is not yet known. Our data showed the inflammatory index was significant but only weakly correlative with age in the study groups, therefore integration with additional measurements was necessary to define more robust predictive immune signatures of age.

Integration of flow cytometry and plasma biomarker data revealed hundreds of immune parameters with weak (coefficients = 0.2–0.4), albeit significant correlations with age, however CD38 expression on T cells had a moderate and inverse correlation with age in HC that was not altered in the HIV-infected group. CD38 is a multi-functional protein that acts as i) an ecto-enzyme (expressed on cell membrane) consuming nicotinamide adenine dinucleotide (NAD+) and ii) a receptor that regulates intracellular calcium. NAD metabolism is a therapeutic target in the aging field, including the sirtuin proteins and the poly-ADP-ribose polymerase (PARP) which are also NAD+ consumers [41, 42]. Much of the work on aging and NAD+ has been performed using mouse models in which CD38 increases on certain cell types with age [43], however differences in tissue expression and receptor signaling have been noted in mice and humans, therefore while it is tempting to connect CD38 expression decline on T cells to NAD+ metabolism and aging in our study, we will focus on the receptor activity and signal transduction downstream of CD38 ligation for which there is more evidence in human T cells [44, 45].

CD38 cooperates with T Cell Receptor/CD3 signaling in lipid rafts as it cannot directly signal through its short cytoplasmic tail. It is highly enriched in cord blood and on Naïve compared to Memory CD4+ T cells [46, 47], which is in line with an overall decline with age as the frequency of Naïve cells decrease throughout life as documented in the FLORAH cohort and by others [7, 12, 48]. CD38 may help amplify signaling through the TCR and the loss of its expression over time could represent a move toward senescence of T cells and the adaptive immune system. Along these lines, in addition to CD38 decline, we observed an increase in immune-senescent CD8 T cells (defined by CD57+CD28-, [49]) with age in HIV-negative and HIV+ participants in this study. In viremic, untreated HIV infection CD38 expression is considered a biomarker for disease progression [50], however during treated HIV infection, such as in the FLORAH cohort, its expression on T cells was not correlated with HIV (i.e., higher frequencies compared to uninfected) [7].

Interestingly, co-expression of CD38 with HLA-DR on T cells is a biomarker for immune activation especially in HIV infection [5], and this biomarker on CD4 memory cells (TM) was included in the IMAP-25 signature but increased with age in PWH only. HLA-DR expressing (CD38-) T cells, especially in the Naïve T cell subset, increased with age in both HIV-negative and HIV+ suggesting increased immune activation of Naïve cells is common in aging and appears to be exacerbated in the context of HIV infection as the PWH demonstrated stronger correlations compared to HC. Another notable and consistent difference observed between the groups was the increase in CD4 Th2 cells with age in HC that was absent in PWH. Though it is controversial, it has been proposed that the switch from Th1-to-Th2 is associated with HIV disease progression [51, 52]. The accumulation of Th2 cells has also been observed with aging in the general population [53], as our data corroborates.

As technological advancements continue to allow for simultaneous collection of many datapoints, strategies for data reduction and focusing on biologically relevant parameters are needed. The statistical strategy we used allowed us to reduce the number of immune features by 50-100-fold. This will be useful for future studies looking to assess immunological age by reducing costs associated with acquiring data from multiple flow cytometry panels and multiplex plasma biomarker detection. The reagent costs for these assays are high as well as the statistical disadvantages when you must account for multiple comparisons.

Cytomegalovirus (CMV) seropositivity was assessed in the FLORAH cohort [19] and plasma IgG titers were included as one of the 27 cell-free analytes included in the model for age. CMV IgG titers have been shown to increase with age in cohorts of HIV-negative populations over 70 years of age [54]. In that study, the authors showed that prior to age 70 the change in titer levels were not found to be significant as age increased. Our cohort has individuals ranging in age from 19 to 77 yrs and we did not find a significant correlation of CMV IgG titer with age in the HIV negative population, however HIV+ individuals both had higher titers than HC and showed a positive correlation with age [19]. Despite this fact, CMV titers were not included in any of the age-prediction models that were generated. A recent study showed that CMV IgG titers correlated with markers of microbial translocation and inflammation in an HIV-infected cohort (ART-naïve and treated), including iFABP [55]. An important limitation of data reduction methods such as Lasso and Elastic Net are that they may remove some biologically relevant parameters from the model if they show strong correlation with selected parameters with the best fit for the current model. Additionally, the model may have been more robust if participants in the octogenarian population and older were included, however given the natural history of the HIV epidemic these older populations are still rare in the HIV-infected community. Despite these limitations, our results propose a group of potential biomarkers that can be used to assess immunological age in diverse clinical settings and provide new therapeutic targets in the fields of Aging and HIV.

## METHODS

### FLORAH study participant characteristics and study design

Study participants were recruited from University of Miami, Jackson Memorial, and VA Hospitals in Miami, FL. HIV-infected, cART-treated participants all demonstrated virus suppression (HIV RNA <40 copies/ml) for at least 1 year prior to enrollment. All participants were administered the seasonal influenza TIV (trivalent inactivated vaccine) and provided peripheral blood samples at pre-vaccination (T0) and postvaccination time points: day 7 (T1), day 21 (T2), and week 24 (T3). Serum titers for Abs against each vaccine strain were determined at every time point by hemagglutination inhibition assay (HAI) as described previously (56). Individual vaccine strain antigens were provided as gifts from Giuseppe del Giudice (Novartis, Siena, Italy). For the 2013–2014 and 2014–2015 seasons the 3 strains in the vaccine were H1N1 A/California/7/2009, H3N2 A/Texas/50/2012, and B/Massachusetts/02/2012-like. For the 2015–2016 season the 3 strains were H1N1 A/California/7/2009, H3N2 A/Switzerland/9715293/2013, and B/Phuket/3073/2013. Peripheral blood mononuclear cells (PBMCs) and plasma were stored in liquid nitrogen and –80°C freezers, respectively, until further experiments were performed.

### Ethics statement

The study was approved by the University of Miami Institutional Review Board. Voluntary signed informed consent was obtained from every participant prior to participating in the study.

### Multiparameter flow cytometry

Previously cryopreserved PBMC were thawed and stained for acquisition of flow cytometry data as described [7]. 6 panels of commercially available, fluorochrome-conjugated monoclonal antibodies (Supplementary Table 2) were assessed for expression on PBMC samples collected at pre-vaccination (T0) using a BD Fortessa instrument. Data were analyzed manually using FlowJo V10 (Tree Star, Inc.).

Some cell subsets discussed in text and shown in figures were abbreviated and defined as follows, divided by cell type. For T cells: TN (Naive, CD45RO-CD27-), TCM (Central Memory, CD45RO+CD27+CCR7+), TTM (Transitional Memory, CD45RO+CD27+CCR7-), TEM (Effector Memory, CD45RO+CD27-), TEFF (Effector, CD45RO-CD27-), pTFH (peripheral T follicular helper, CXCR5+ TCM), TH1 (CXCR3+CCR6-), TH2 (CXCR3-CCR6-), TH17 (CXCR3-CCR6+), TH1-TH17 (CXCR3+CCR6+). For B cells: AM (B cell Activated Memory, CD19+IgD-CD27+CD21-), RM (B cell Resting Memory, CD19+IgD-CD27+CD21+), DN (B cell Double Negative, CD19+IgD-CD27-CD21-).

### Measurement of cell-free plasma markers

Proteins from plasma were measured from FLORAH participants as described previously [7].

### Statistical methods

Inflammatory Index: Backward stepwise regression was applied to plasma biomarker data from 26 individual markers to identify a group of markers with the strongest associations with age. Then, PCA was performed using the resulting 8 plasma biomarkers and the Inflammatory Index was calculated for each individual as the sum of the standardized variables (8 plasma markers) multiplied with their weights extracted from PC1.

### Age prediction

After data preprocessing, 209 participants with complete information on 1357 markers (1330 immunological markers, 26 cytokines and CMV IgG) were subject to building aging prediction model. First, spearman correlation and multivariate regression analysis were performed using immune markers to identify significant markers associated with age, then a data reduction approach by Lasso or Elastic Net Regression was used to further select the highly correlated variables. Using 4 machine learning algorithms (Linear regression, Neural network, Decision Forest regression, boosted decision tree) we found Linear regression generated the best prediction.

### Build aging model

Fit generalized linear model via penalized maximum likelihood at alpha parameter 0, 0.05, 0.1…1. Repeated-corrected 10-fold CV (cross-validation) was run for 500 times and minimum averaged RMSE (Root Mean Squared Error) was used to select optimal regularization parameter alpha. The resulting model from Lambda1se (the value of λ that gives one standard error away from the minimum error) was further assessed by bootstrapping 500 times. The candidate markers included only markers present more than 250 times.

### Optimizing aging model

Initial aging model was tuned to get best prediction on age using Microsoft Azure Machine Learning Studio. Model tuning was performed on 70% train and 30% test in HC and HIV separately. Cross-validation was performed by 5 or 10 folds. Markers were evaluated by their permutation feature importance. Optimal model was selected by minimum averaged RMSE and highest Coefficient of Determination (R-squared). Optimal model was further tuned on 4 machine learning algorithms (Linear regression, Neural network, Decision Forest regression, boosted decision tree). Linear regression generated best prediction.

### Aging rate

Correlation of predicted age (Age predicted by immunological markers, aka “immunological age”) and age was performed by Spearman correlation analysis. Difference of aging in HC and HIV (Aging Rate and Age Advancement) was identified by Two Sample *t*-test or Welch Two Sample *t*-test on predicted age by combined aging model.

## Supplementary Materials

Supplementary Figure 1

Supplementary Tables 1-2 and 5

Supplementary Tables 3 and 4
