# Supplementary material for: Immunological age prediction in HIV-infected, ART-treated individuals
Source: Aging (Albany NY). 2021 Oct 11;13(19):22772–91. doi: 10.18632/aging.203625 (PMC8544329; doi:10.18632/aging.203625)
Supplement: Supplementary Figure 1 [file aging-13-203625-s001.pdf]

SUPPLEMENTARY FIGURE

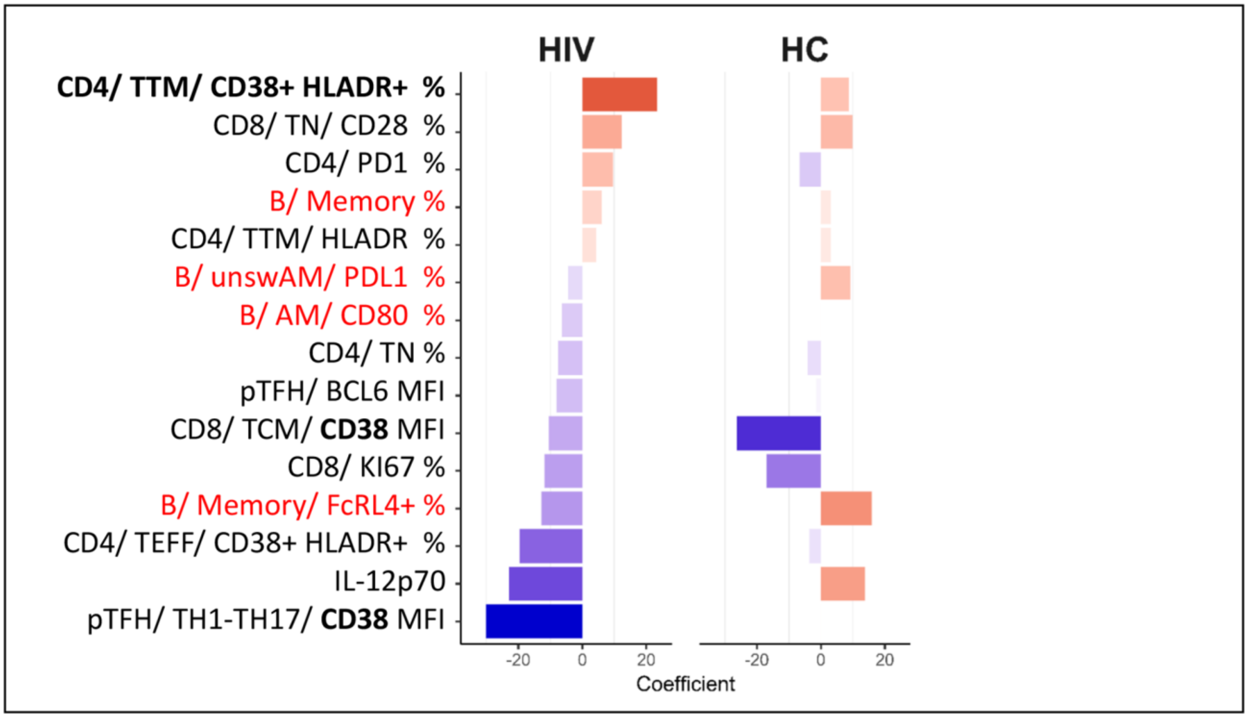

Supplementary Figure 1. Prediction modeling for chronological age in HIV-positive population. Bar graph shows each of the 15 parameters included in the HIV+ trained model and indicates the coefficient for each parameter when applied to HIV+ participants (left) and HIV-negative, healthy controls (HC, right). Predictive accuracy in HIV+ participants was 55.48%. Red bars denote a positive association with predicted age and blue bars denote a negative association. Red font indicates non-T cell parameters and bold font indicates CD38-containing parameters.
