# Supplementary material for: Immunological age prediction in HIV-infected, ART-treated individuals
Source: Aging (Albany NY). 2021 Oct 11;13(19):22772–91. doi: 10.18632/aging.203625 (PMC8544329; doi:10.18632/aging.203625)
Supplement: Supplementary Tables 1-2 and 5 [file aging-13-203625-s002.pdf]

SUPPLEMENTARY TABLES

Supplementary Table 1. Cell-free analytes measured in plasma.

Marker Name
BNP
CMV IgG
CRP
CXCL13
D-Dimer
Intestinal fatty acid binding protein (iFABP)
Interferon (IFN)-a
Interferon-g
Interleukin (IL)-10
Interleukin-12p70
Interleukin-17A
Interleukin-1B
Interleukin-2
Interleukin-21
Interleukin-6
Interleukin-8
LPS
MCP1
Neopterin
Soluble CD14
Soluble CD163
Soluble CD25
Soluble ICAM
Soluble TNFRI
Soluble TNFRII
Soluble VCAM
TNF

Supplementary Table 2. Multiparameter flow cytometry panels used to obtain immune-phenotypic data.

Fluorochrome/ Channel	Immune Activation Panel	Immuno-regulatory Panel	Immune Checkpoint Panel	T helper subset Panel	B cell Panel	Monocyte/ NK Panel
BUV395	CD3	CD3	CD3	CD3	CD3	CD3
BV421	ICOS	BCL6	CD57	CCR4	CD71	PDL1
BV605	CXCR3	CD127	PD1	CXCR3	CD80	CD56
BV650	PD1	PD1	CD4	PD1	PDL1	CD11b
BV711	Ki-67	CD25	TBET	CD95	Ki-67	Inv NKT Cell
AQUA	Live/Dead	Live/Dead	Live/Dead	Live/Dead	Live/Dead	Live/Dead
APC/AF647	CXCR5	CXCR5	EOMES	CXCR5	FCRL4	CD16

APC-Cy7/APCH7	CD45RO	CD45RO	CD45RO	CD45RO	CD38	HLA-DR
AF700	CD8	CD8	CD8	CD8	CD20	CD8
PE	CD38	FOXP3	TIM3	CCR5	IL-21R	CX3CR1
ECD/PE-CF594	CCR7	CCR7	CCR7	CCR7	CD138	CCR7
PE-Cy5	CD27	CD4	CD28	CD27	CD21	CD45
PE-Cy7	CCR6	CTLA-4	LAG3	CCR6	CD10	CD14
FITC/AF488	HLA-DR	CD39	2B4	CD161	IgD	CCR2
PerCP-Cy5.5	CD4	Ki-67	TIGIT	CD4	CD27	CD4

Please browse Full Text version to see the data of Supplementary Tables 3 and 4.

Supplementary Table 3. Spearman correlation results for univariate analysis of parameter with age in HC.

Supplementary Table 4. Spearman correlation results for univariate analysis of parameter with age in HIV.

Supplementary Table 5. Overlapping age-associated parameters in HIV-negative and HIV-positive groups: Same direction parameters (full list). Related to Figure 3.

Immune Parameter	Coefficient_HC	Coefficient_HIV
CD8/TCM/CD38- HLADR+ %	0.376	0.381
CD8/TN/CD28 %	0.357	0.228
CD8/CD38- HLADR+ %	0.344	0.432
CD4/TN/2B4 %	0.327	0.264
CD8/TN/CD38- HLADR+ %	0.307	0.443
CD8/TCM/PD1 MFI	0.307	0.37
CD4/TN/CD38- HLADR+ %	0.298	0.424
CD8/TN/TIGIT %	0.286	0.257
CD4/TEFF/CCR5 MFI	0.283	0.197
CD4/TEM	0.263	0.293
CD8/TN/CD57 %	0.251	0.344
sCD163	0.231	0.259
CD4/CD38- HLADR+ %	0.229	0.499
Inflammatory Mono	0.221	0.338
pTFH/TH2/CD38- HLADR+ %	0.202	0.372
CD8/TEFF/CD57 %	0.201	0.256
Transitional B cells/PDL1 %	0.201	0.221
CD4/TCM/NONTFH/TH1-TH17/CD38+ HLADR- %	–0.201	–0.296
CD8/TTM/CD38 MFI	–0.206	–0.248
CD4/TCM/NONTFH/TH17/CD38 %	–0.207	–0.245
CD8/TCM/CD38+ HLADR+ %	–0.209	–0.212
B/Double Neg/IL-21R+ %	–0.21	–0.207
CD4/TEFF/CD38 %	–0.217	–0.282
CD4/TCM/NONTFH/TH17/CD38+ HLADR- %	–0.229	–0.381
CD4/TN/ICOS %	–0.237	–0.24

CD8/TEFF/CD38+ HLADR- %	–0.238	–0.313
pTFH/TH1-TH17/ICOS %	–0.242	–0.291
CD4/TCM/NONTFH/TH17/ICOS %	–0.243	–0.243
CD4/TCM/ICOS %	–0.248	–0.217
CD4/TCM/NONTFH/TH17/CD38 MFI	–0.255	–0.257
CD8/TEM/CD38+ HLADR- %	–0.255	–0.339
CD4/TCM/NONTFH/ICOS %	–0.256	–0.204
pTFH/TH1/CD38+ HLADR- %	–0.257	–0.26
CD4/TEM/ICOS %	–0.263	–0.313
CD4/TEM/CD38+ HLADR- %	–0.269	–0.294
CD4/TEFF/CD38+ HLADR- %	–0.273	–0.351
CD4/TCM/NONTFH/TH1/CD38 MFI	–0.292	–0.262
CD4/TCM/NONTFH/TH1/CD38 %	–0.293	–0.221
CD4/TCM/NONTFH/TH1/CD38+ HLADR- %	–0.296	–0.292
pTFH/TH2/ICOS %	–0.298	–0.275
CD4/TCM/NONTFH/TH2/CD38+ HLADR- %	–0.304	–0.347
CD8/TN/CD38 %	–0.308	–0.28
CD4/TN	–0.31	–0.341
pTFH/ICOS %	–0.312	–0.244
CD8/TEM/CD38 MFI	–0.313	–0.225
CD4/TN/CD38+ HLADR- %	–0.317	–0.247
CD4/TCM/NONTFH/TH2/CD38 %	–0.317	–0.29
CD4/TN/CD38 MFI	–0.318	–0.21
CD8/TCM/KI67	–0.34	–0.253
pTFH/TH17/ICOS %	–0.34	–0.336
CD4/TCM/NONTFH/TH2/CD38 MFI	–0.343	–0.29
pTFH/TH17/CD38 %	–0.347	–0.244
CD8/TEM/CD38 %	–0.35	–0.214
CD4/TCM/NONTFH/CD38+ HLADR- %	–0.351	–0.324
CD8/TN/CD38+ HLADR- %	–0.365	–0.38
pTFH/TH1-TH17/CD38+ HLADR- %	–0.367	–0.216
pTFH/TH17/CD38+ HLADR- %	–0.371	–0.328
CD4/TCM/NONTFH/CD38 %	–0.376	–0.234
CD8/TN/CD38 MFI	–0.38	–0.357
CD4/CD38+ HLADR- %	–0.386	–0.394
CD4/TCM/NONTFH/CD38 MFI	–0.388	–0.229
CD4/CD38 %	–0.396	–0.335
pTFH/CD38+ HLADR- %	–0.398	–0.294
pTFH/TH2/CD38+ HLADR- %	–0.402	–0.287
CD8/CD38 %	–0.402	–0.305
CD4/TCM/CD38+ HLADR- %	–0.419	–0.342
CD4/CD38 MFI	–0.422	–0.354

CD4/TCM/CD38 (%)	–0.424	–0.216
pTFH/TH17/CD38 MFI	–0.428	–0.273
pTFH/TH2/CD38 MFI	–0.431	–0.235
CD4/TTM/CD38+ HLADR- %	–0.443	–0.337
pTFH/CD38 MFI	–0.453	–0.214
CD8/CD38 MFI	–0.464	–0.361
CD4/TCM/CD38 MFI	–0.465	–0.232
CD8/CD38+ HLADR- %	–0.469	–0.447
CD8/TCM/CD38 %	–0.537	–0.353
CD8/TCM/CD38+ HLADR- %	–0.555	–0.4
CD8/TCM/CD38 MFI	–0.559	–0.383
